# Case report: New subtype of gastric adenocarcinoma arising from an *H. pylori* infection-negative stomach: Foveolar epithelium and mucous neck cell-mixed type adenocarcinoma

**DOI:** 10.3389/fonc.2022.970231

**Published:** 2022-08-29

**Authors:** Xuemei Liu, Xinglong Wu, Jiaxing Zhu, Kazuyoshi Yagi, Yoichi Ajioka, Shuji Terai, Kenichi Mizuno, Hongping Li, Biguang Tuo, Lianjun Di

**Affiliations:** ^1^ Department of Gastroenterology, Digestive Disease Hospital, Affiliated Hospital of Zunyi Medical University, Zunyi, China; ^2^ Department of Pathology, Affiliated Hospital of Zunyi Medical University, Zunyi, China; ^3^ Uonuma Institute of Community Medicine, Graduate School of Medical and Dental Sciences, Niigata University, Niigata, Japan; ^4^ Division of Molecular and Diagnostic Pathology, Graduate School of Medical and Dental Sciences, Niigata University, Niigata, Japan; ^5^ Division of Gastroenterology & Hepatology, Graduate School of Medical and Dental Sciences, Niigata University, Niigata, Japan

**Keywords:** *H. pylori* infection-negative stomach, new subtype of gastric cancer, mixed foveolar epithelium and mucous neckcell-type adenocarcinoma, bidirectional differentiation, rareness

## Abstract

The characteristics of *Helicobacter pylori* (*H. pylori*) infection-negative gastric cancer have not been well documented because of its rarity, despite several types of *H. pylori* infection-negative gastric cancers being reported. In this report, we describe a case of early gastric cancer that developed without *H. pylori* infection with characteristic magnifying narrow-band imaging and novel histological findings. The difficulty in making an accurate diagnosis and differential diagnosis is highlighted, with the goal of providing more clinical experience for the diagnosis of *H. pylori* infection-negative gastric cancer.

## Introduction

*Helicobacter pylori* (*H. pylori*) infection causes chronic atrophic gastritis and progresses to gastric cancer (GC) (1), which is considered one of the most serious healthcare problems worldwide (2). Previously, the prevalence of *H. pylori* infection-negative gastric cancer (HPINGC) was very low, at 0.42%–5.4% of all cases of GCs (3–5). However, *H. pylori* eradication therapy is now becoming widespread, and the morbidity of *H. pylori* infection is decreasing. Therefore, it is expected that HPINGC will become a comparatively more common disease in the near future. Over the last 10 years, several types of HPINGC, including intraepithelial signet ring cell carcinoma and fundic gland type, foveolar epithelial type, cardiac gland type, pyloric gland type, and mixed type GC, classified by the Mizutani group, have been reported (3–5). Herein, we report a case of a 48-year-old woman with a new subtype of gastric adenocarcinoma: mixed foveolar epithelium and mucous neck cell-type adenocarcinoma in *H. pylori* infection-negative mucosa.

## Case report

A 48-year-old woman visited our hospital after experiencing epigastric pain for approximately 1 month. There was no obvious cause of the paroxysmal dull abdominal pain and no history of loss of appetite, postprandial vomiting, or gastrointestinal bleeding. The physical examination was normal. The ^14^C urea breath test (UBT) and serum *H. pylori* antibody results were negative, and she had no history of *H. pylori* eradication. Routine laboratory investigations were unremarkable.

**Figure 1:**
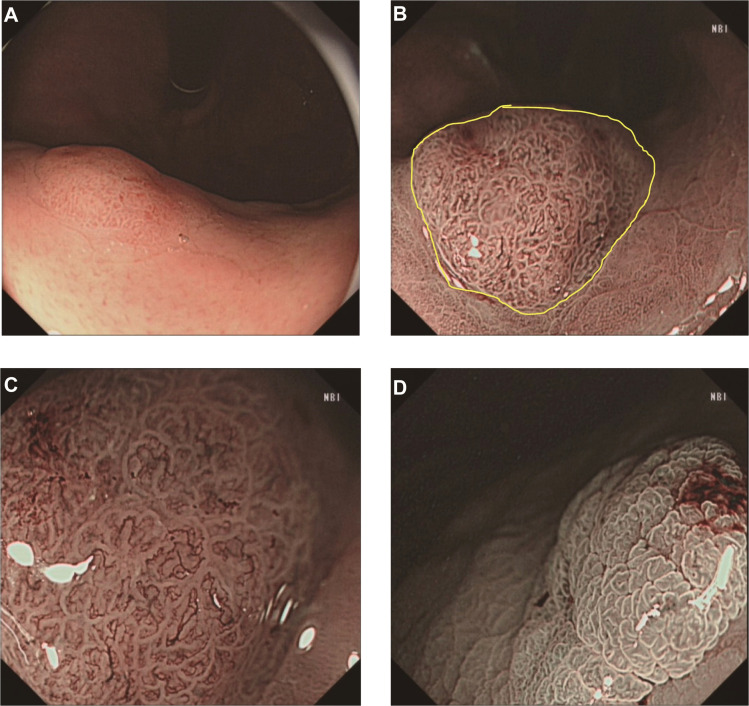
Endoscopic images. Esophagogastroduodenoscopy showed a yellowish and elevated lesion in the upper part of the greater curvature of the *H. pylori*-negative background **(A)**. NBI showed a defined light brownish elevated area **(B)**. Magnifying narrow-band imaging revealed an expanded and thinned white zone with changed polarity, as well as dilated and irregular microvascular architecture. The granular microsurface structure of this lesion was stained with acetic acid **(D)**. The yellow dotted line indicates the extent of the lesion diagnosed as cancer by histopathology.

**Figure 2:**
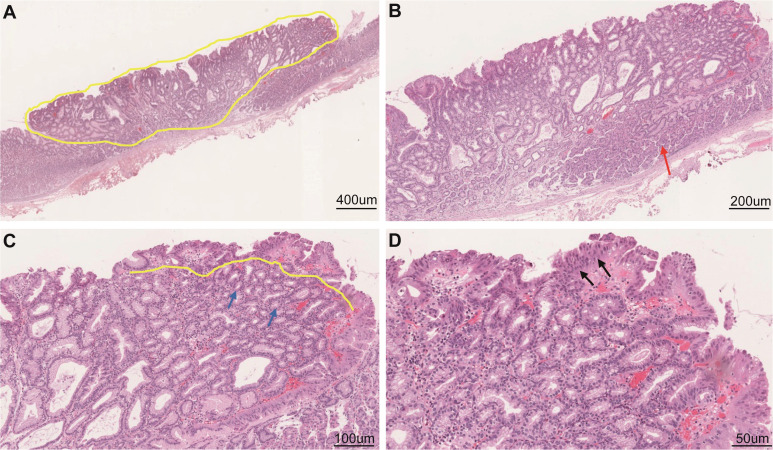
Histological examination of the resected specimen. Low-power field **(A)** and high-power field **(B–D)** of hematoxylin and eosin staining. Tumor cells were similar to mucous neck cells **(C)** and foveolar epithelium **(D)**. Scale bars represent 400 μm **(A)**, 200 μm **(B)**, 100 μm **(C)**, and 50 μm **(D)**. The yellow dotted line indicates the extent and size of the diagnosed cancer; the red arrow indicates a normal gastric corpus gland; the black arrows indicate sufficiently high cellular atypia in the surficial part; and the yellow line and blue arrows indicate that the undersurface part is the cancerous lesion.

**Figure 3:**
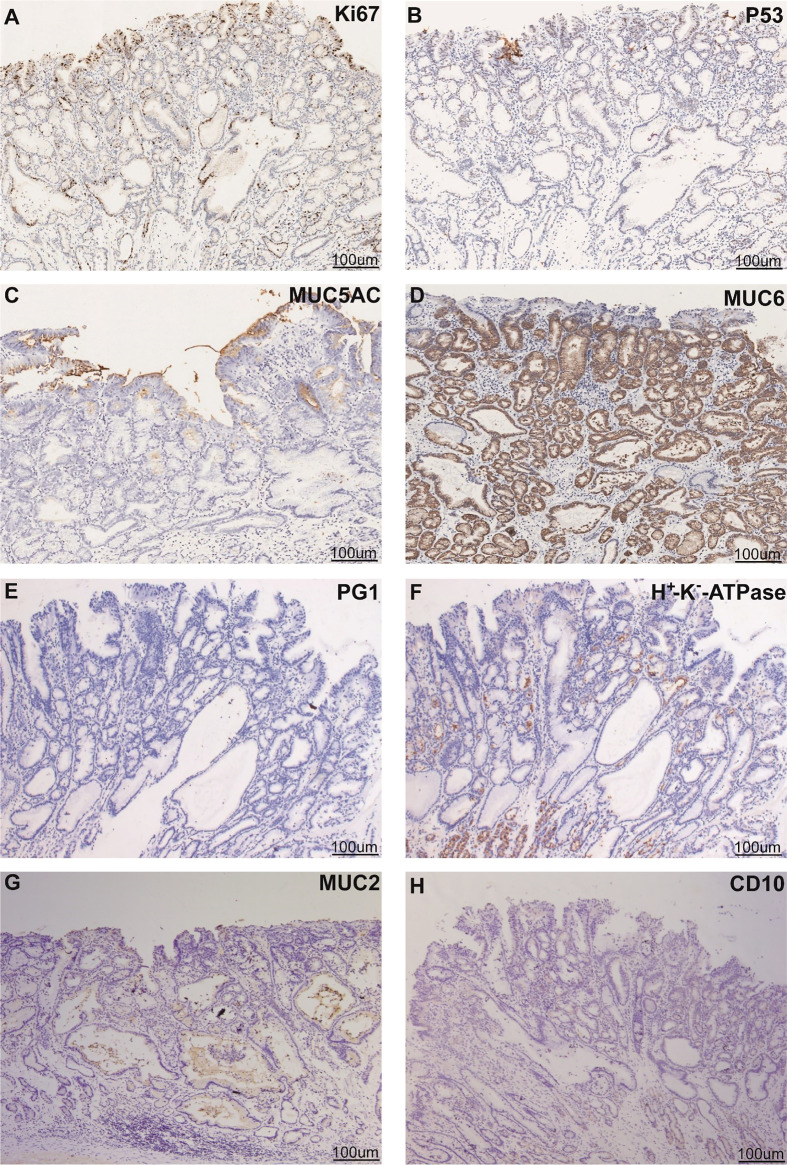
Phenotypic marker expression by immunohistochemistry staining with Ki67 **(A)**, P53 **(B)**, MUC5AC **(C)**, MUC6 **(D)**, PG1 **(E)**, proton pump (H^+^ -K^+^-ATPase) **(F)**, MUC2 **(G)**, and CD10 **(H)**. Scale bars represent 100 μm. Black arrows indicate different positively stained parts.

**Figure 4:**
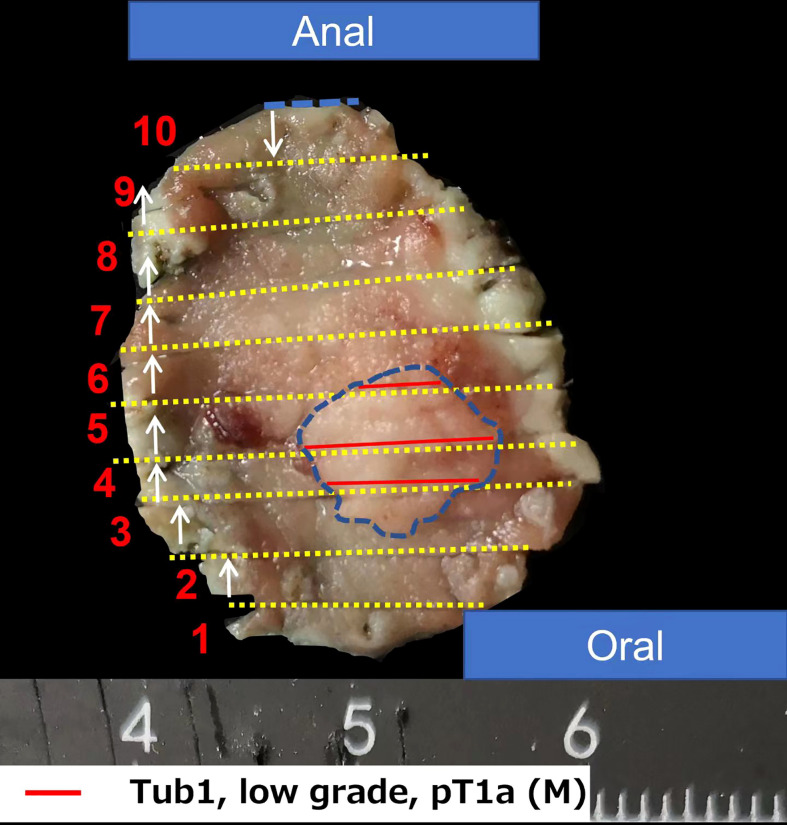
Pathological recovery diagrams. New subtype of gastric adenocarcinoma arising from an *H. pylori*-uninfected stomach: foveolar epithelium and mucous neck cell-mixed type adenocarcinoma, pT1a (M), Ly0, V0, pUL0, pHM0, and pVM0. The specimen was 2 × 2.5 cm and type 0-IIa, and the cancerous lesion was 7 × 5 mm with red marking in the blue circle.

Esophagogastroduodenoscopy showed a yellow and elevated lesion in the upper part of the greater curvature, and the background mucosa showed a regular arrangement of collecting venules (RAC), which was specific to the normal corpus mucosa with no atrophic changes (**Figure 1A**). The lesion presented as a defined light brownish area under narrow-band imaging (NBI) (**Figure 1B**). Magnifying endoscopy with NBI (ME-NBI) revealed an expanded and thinned white zone with altered polarity and a dilated and irregular microvascular architecture (**Figure 1C**). The granular microsurface structure was detected by acetic acid staining, which was completely different from the surrounding fundic gland mucosa (**Figure 1D**). The initial suspected diagnosis was *H. pylori*-negative early GC based on all the above observations. This elevated lesion was diagnosed as differentiated gastric mucosal cancer that was 2 cm in diameter without ulceration or deep invasion signs, was defined as cT1a(M) by the eCura system, and met endoscopic submucosal dissection (ESD) indications (6). Therefore, diagnostic ESD was performed. Histopathologic examination of the resected specimen showed that the surrounding mucosa of the elevated lesion was a normal gastric fundic gland (**Figures 2A,B**); however, the superficial part of the elevated lesion had cellular atypia, and the nuclei were large and hyperchromatic with multiple layers (**Figures 2A–D**). These cells were Muc5AC positive (**Figure 3C**). Furthermore, under the atypia surface, tumor cells showed a pyloric gland-like morphology (**Figures 2C, D**), which was positively stained for MUC6 but negatively stained for PG1 and H^+^-K^+^-ATPase. Both layers, namely, the superficial part and the undersurface, displayed negativity for MUC2 and CD10 (**Figures 3D–H**). The Ki-67 labeling index was high in the superficial part and low in the undersurface, and P53 was positive in the superficial part (**Figures 3A, B**). Therefore, the final pathological diagnosis was early mixed foveolar epithelium and mucous neck cell-type gastric adenocarcinoma that was 7 × 5 mm in size (**Figure 4**). The postoperative course was uneventful, and she has been asymptomatic with no recurrence for 18 months.

## Discussion

Currently, the prevalence of *H. pylori* infection is gradually decreasing with improvements in living conditions and eradication therapy, causing a decline in *H. pylori* infection-related GC. However, the frequency of HpNGC may show a relative increase. HpNGC is an adenocarcinoma that occurs in the stomach without active or previous *H. pylori* infection. Although various types of HpNGC have been reported (3–5), the main causal factors and diagnostic criteria have not yet been definitively established. Therefore, it is essential to recognize different types of HpNGC.

First, the definition of *H. pylori* infection negativity should be determined. Endoscopic, pathological, and clinical assessments are recommended as the criteria for the diagnosis of the gastric mucosa background without *H. pylori* infection, including negativity for *H. pylori* by endoscopic or pathological findings, negative UBT or serum IgG test, and no *H. pylori* eradication history. For endoscopic diagnosis, the lack of mucosal atrophy and the presence of RAC indicate *H. pylori* negativity. Additionally, linear erythema and fundic gland polyps are included as signs of *H. pylori* infection negativity. With pathological methods, biopsy specimens taken from the stomach are evaluated according to the updated Sydney System by pathologists. Combining endoscopic detection with pathological findings can diagnose not only current *H. pylori* infection but also any past infection by evaluating atrophic changes and intestinal metaplasia (7, 8). With clinical methods, *H. pylori* negativity is confirmed by two or more tests, such as the UBT, serum or urine antibody test, and stool antigen test, because each test may give a false-negative result for *H. pylori* infection. Clinical methods can diagnose current infection with *H. pylori* but cannot distinguish between infection-naïve patients and patients with past infection (9). Together, the minimum criteria for *H. pylori* negativity require negative findings from two or more methods that include endoscopic or pathological findings and negative UBT or serum IgG tests, as well as no eradication history.

Previously, six types of HpNGC were reported, namely, intraepithelial signet ring cell carcinoma (SRCC), fundic gland type, foveolar epithelial type, cardiac gland type, pyloric gland type, and mixed type GC (3–5). However, the variety of cells of origin may account for their varied appearance. Generally, SRCC presents as a discolored flat or depressed lesion in the lower or middle part of the stomach in relatively young female patients (5). Compared with SRCC in *H. pylori* infection-positive stomach tissue that presents in all layers, *H. pylori*-negative SRCC is generally located in the proliferative zone, as its pathological growth pattern has less invasion (10). The fundic gland type is usually located in the middle and upper part of the stomach in comparably older patients, and the predominant gross type is submucosal tumor-like. ME-NBI shows arborizing-like vessels and dilated crypts, which are defined as differentiated-type adenocarcinoma with chief cell differentiation that positively stains for H ^+^/K ^+^ ATPase, a parietal cell marker, and/or pepsinogen-I, a chief cell marker (11). The foveolar epithelial type is believed to develop from surface mucosal cells, and the gross type consists of lateral spread with some degree of elevation, villous or papillary surfaces, and a raspberry-like appearance of the non-atrophic gastric mucosa (12, 13). Unlike the undifferentiated type or fundic gland type, the field of cancerization is superficial, resulting in the appearance of defined demarcation, with MUC5AC positivity by immunohistochemistry (IHC). The cardiac gland type appears to originate from the cardiac gland, which displays depressed and red lesions, and perturbed ductal structures are observed in NBI-ME. The pyloric gland type is derived from the pyloric region with positive staining for CD10 and chromogranin A. The mixed type is believed to originate from various cell types, including foveolar epithelial cells, fundic gland cells, pyloric gland cells, or intestinal-type cells.

In this case, the tumor was located in the upper gastric corpus and developed in the fundic gland mucosa without *H. pylori* infection (**Figure 1**). The gross type as well as the microstructure and microvascular architecture in ME-NBI were similar to those of normal GC. Therefore, the final judgment was strongly dependent on the histological examination and IHC analysis. Histologically, the superficial part of the lesion had MUC5AC-positive staining, showed sufficiently high cellular atypia, and appeared smoothly connected to the lower part of the lesion with MUC6 positivity. However, the whole lesion was stained negatively for CD10 and MUC2. Thus, the final diagnosis was gastric-type adenocarcinoma. It is known that tumor cells have the ability to mimic the morphology and function of mucosal epithelial cells where they co-occur. This adenocarcinoma is characterized by its existence above the fundic glands and composition of chief cells, parietal cells, and mucous neck cells. Tumors continuously occur from these mucous neck cells and form MUC6-positive parts. Furthermore, it develops to the foveolar part and forms MUC5AC-positive parts. Therefore, the whole cancerous lesion showed bidirectional differentiation, a foveolar-type epithelium to the surface, and mucous neck cells to the bottom. Since this bidirectional differentiation is a basic characteristic of the gastric mucosa, this tumor merely imitated this characteristic. This structure is different from the previously reported HpNGC.

In addition, this lesion is necessary to distinguish it from adenocarcinoma arising from pyloric gland adenoma. First, this lesion does not show typical endoscopic and histological findings of adenocarcinoma arising from pyloric gland adenoma (14, 15). The cancerous area is a superficial epithelial area showing MUC5AC. The subepithelial component of pyloric adenoma shows low atypical glands as adenoma, which indicates MUC6. A very important issue is that MUC6^+^ glands are not only related to pyloric glands but also associated with mucous neck cells of fundic glands. In this case, tumors developed in the fundic gland mucosa without *H. pylori* infection, and it is more reasonable to regard MUC6^+^ cells as mucous neck cells than those of the pyloric gland. Therefore, this case shows high atypical glands as cancer in the subepithelial MUC6 component. This lesion does not belong to adenocarcinoma arising from pyloric gland adenoma. Therefore, we propose the new entry of this cancer as HpNGC.

In summary, we report a rare case of early GC in an *H. pylori* infection-negative patient with the following characteristic clinicopathological findings: (1) elevated shape with an expanded and thinned white zone, as well as a dilated and irregular microvascular architecture in ME-NBI, and (2) histological differentiation toward mixed foveolar and mucous neck cell mucosa. In the future, the accumulation of cases may clarify the clinicopathological characteristics, but the current study does seem to describe a novel type of HpNGC.

## Data availability statement

The original contributions presented in the study are included in the article/supplementary material. Further inquiries can be directed to the corresponding authors.

## Ethics statement

The studies involving human participants were reviewed and approved by the Ethics Committee of Zunyi Medical University [2020] 2-515. The patients/participants provided their written informed consent to participate in this study. Written informed consent was obtained from the individual(s) for the publication of any potentially identifiable images or data included in this article.

## Author contributions

Analysis of the results and the writing of the manuscript: XL. Collection of data: XL, KY, YA, ST, KM, and HL. Analysis of the results and pathological pictures: XW and JZ. Design and implementation of the research and revision of the manuscript: BT and LD. All authors contributed to the article and approved the submitted version.

## Funding

This research was supported by the National Natural Science Foundation of China (81860103 and 82070536 to XL and 81572438 to BT) and the Guizhou Province International Science and Technology Cooperation (Gastroenterology) Base (Qian Ke He Platform Talents-HZJD [2021] 001 to XL).

## Conflict of interest

The authors declare that the research was conducted in the absence of any commercial or financial relationships that could be construed as a potential conflict of interest.

## Publisher’s note

All claims expressed in this article are solely those of the authors and do not necessarily represent those of their affiliated organizations, or those of the publisher, the editors and the reviewers. Any product that may be evaluated in this article, or claim that may be made by its manufacturer, is not guaranteed or endorsed by the publisher.
